# Eggshell membrane and its major component lysozyme and ovotransferrin enhance the secretion of decorin as an endogenous antifibrotic mediator from lung fibroblasts and ameliorate bleomycin-induced pulmonary fibrosis

**DOI:** 10.1016/j.bbrep.2024.101806

**Published:** 2024-08-12

**Authors:** Eri Ohto-Fujita, Miho Shimizu, Aya Atomi, Hiroki Hiruta, Ryota Hosoda, Shinya Horinouchi, Shinya Miyazaki, Tomoaki Murakami, Yoshihide Asano, Yukio Hasebe, Yoriko Atomi

**Affiliations:** a Material Health Science, Graduate School of Engineering, Tokyo University of Agriculture and Technology (TUAT), Tokyo, 184-8588, Japan; b Cooperative Dep. Veterinary Medicine, Tokyo University of Agriculture and Technology, Tokyo, 183-8509, Japan; c Department of Dermatology, Tohoku University Graduate School of Medicine, Sendai, 980-8574, Japan; d Almado, Inc., Tokyo, 104-0031, Japan

**Keywords:** Decorin, Eggshell membrane, Lysozyme, Ovotransferrin, Pulmonary fibrosis

## Abstract

Aging is a high-risk factor for obstructive and fibrotic lung diseases. Fibrotic lung disease leading to decreased lung function is characterized by interstitial remodeling and tissue scarring (sclerosis), with destruction of alveoli and excess deposition of type I collagen, an extracellular matrix component secreted by fibroblasts. Therefore, regulating transforming growth factor-β (TGF-β) as a profibrotic signal is essential to suppress pulmonary fibrosis. In pulmonary fibrosis, TGF-β signaling is mediated by Smad and YAP/TAZ, and TAZ linked to the pathology of pulmonary function is observed in lung fibroblasts from patients with idiopathic pulmonary fibrosis. Although fibrosis is thought to be irreversible, it is an interventional condition. Decorin (DCN) blocks TGF-β signaling in pulmonary fibrosis, although there are no cellular pharmacological methods to stimulate DCN secretion. We previously showed that chicken eggshell membrane (ESM, a well-known wound-healing material) promotes *dcn* gene expression in fibroblasts. In this study, we investigated whether ESM stimulates DCN secretion as an endogenous mediator and ameliorates pulmonary fibrosis. Decorin secretion was significantly enhanced in the WI-38 lung fibroblast culture supernatants supplemented with ESM. This effect was increased with major component lysozyme and maximally promoted in experiments with lysozyme and ovotransferrin (the two main proteins in soluble ESM) at a 16:1 concentration ratio, the ratio in the ESM extract. Decorin secretion by ESM modulates TGF-β signaling in lung fibroblasts by reducing TAZ and pSmad2 nuclear localization. Decorin siRNA experiments confirmed that nuclear localization of TAZ is DCN-dependent. In a mouse model of bleomycin-induced pulmonary fibrosis, all fibrotic markers of ESM treatment group such as hydroxyproline (a collagen deposition marker), and both evaluation of fibrosis density by automated thresholding of picrosirius red-stained lung tissue scan images and Ashcroft fibrosis scores, and also the nuclear localization of TAZ were reduced after 2 weeks compared with control group. Furthermore, long-term (22 week) ESM consumption by healthy individuals significantly improved vital capacity and the forced expiratory volume in 1 s to forced vital capacity ratio (FEV1/FVC). This study reveals that ESM, a well-established wound-healing material, may be a potential preventive medicine for pulmonary fibrosis.

## Highlights

• An antifibrotic decorin suppressed TAZ translocation by TGF-β in WI-38 cells.
• Eggshell membrane and lysozyme promoted decorin secretion from WI-38.
• Eggshell membrane pretreatment inhibited profibrotic TAZ translocation in WI-38.
• 2 weeks eggshell membrane intake of bleomycin mouse ameliorated pulmonary fibrosis.
• 22-weeks long-term ingestion by healthy human significantly improved lung function.

## Abbreviations

BLM: Bleomycin
COL1: Type I collagen
DCN: Decorin
ECM: Extracellular matrix
ESM: Eggshell membrane
FEV1/FVC: Forced expiratory volume in 1 s to forced vital capacity ratio
HDF: High-density frequency
IPF: Idiopathic pulmonary fibrosis
LYZ: Lysozyme
TAZ: Transcriptional coactivator with PDZ-binding motif
TF: Ovotransferrin
TGF-β: Transforming growth factor-β
VC: Vital capacity
YAP: Yes-associated protein

## 1 Introduction

Respiratory function declines with age, accompanied by structural and functional changes in the lungs and decreased function of the diaphragm and other respiratory muscles [1]. Age also increases the risk of primary lung cancer, respiratory infections, and obstructive and fibrotic lung disease [2]. Fibrosis is a disease that harms the extracellular environment and can invade all organs, accounting for 45 % of deaths in developed countries [3]. Fibrotic lung disease is characterized by interstitial remodeling, destruction of the tissue structure, irreversible scarring, and decreased lung function. Organ stiffness is regulated by the extracellular matrix (ECM) secreted from cells. Since downregulation of type I collagen (COL1) is key to inhibiting fibrosis, it is important to regulate transforming growth factor (TGF)-β as an upstream chemical signal [4].

Decorin (DCN) is also known as an antifibrotic proteoglycan in lung fibroblasts [5,6] and suppresses TGF-β-dependent fibrotic signaling by binding to and sequestering TGF-β [7]. Mice with bleomycin (BLM)-induced pulmonary fibrosis transfected with DCN expression vector show reduced fibrosis [5]. However, there are no pharmacological methods to stimulate DCN secretion. TGF-β signaling is also mediated by Smad and YAP/TAZ [[8], [9], [10]], with increased nuclear staining of YAP/TAZ in lung fibroblasts of patients with idiopathic pulmonary fibrosis (IPF) [9,10]. Additionally, nuclear localization of TAZ is more evident in stiff matrices related to the physiological hallmarks of increased lung stiffness in IPF [9,10].

The eggshell membrane (ESM) is a well-established wound-healing material [11] that is attracting attention as a new biomaterial with nutritional and pharmacological applications, including wound healing [12]. We showed that ESM promotes *dcn* expression in human dermal fibroblasts [13]. Human dermal fibroblasts increase the expression of ECM genes such as *dcn*, which is essential for proper fibrillogenesis of COL1 by ESM covalently bound to the artificial cell membrane biointerface using 2-methacryloyloxyethyl phosphorylcholine (PMBN) [13]. Oral administration of ESM to rats improves liver fibrosis [14]. Proteomic analysis shows that ESM is a complex material consisting of over 500 proteins [15], although component-activity relationships other than antioxidants [16] were not explored. Lysozyme (LYZ) [17] and ovotransferrin (TF) [18] are the two major matrix protein components with therapeutic interests.

Recently, we reported that oral supplementation with ESM for 8 weeks in healthy subjects significantly increased respiratory function (forced expiratory volume in 1 s to forced vital capacity ratio [FEV1/FVC]) compared to that in controls [19], demonstrating the possible therapeutic use of ESM targeting lung health. The purpose of this study was to examine the effects of ESM on pulmonary fibrosis by inducing *dcn* expression and suppressing pulmonary fibrosis signaling.

## 2 Materials and methods

### 2.1 In vitro cell studies

WI-38 lung fibroblasts (passage 4–7, 2 × 10^4^ cells) were seeded overnight on 22 × 22 mm^2^ coverslips. For treatment with TGF-β1, the culture medium was replaced with 2 mL of fresh medium without serum. WI-38 cells were incubated for 2 h before TGF-β1 addition with hydrolyzed ESM, (Almado, Tokyo, Japan), LYZ (L6876-5G, Sigma Aldrich, St. Louis, MO, USA), or TF (C0755-100 MG, Sigma Aldrich, St. Louis, MO, USA). Cells were treated with 5 ng/mL recombinant human TGF-β1 (Abcam, Cambridge, UK) for 5 h and culture supernatants were collected before fixing the cells for DCN quantification using Decorin DuoSet enzyme linked immunosorbent assay (ELISA) (#DY143; R&D Systems, Minneapolis, MN, USA). Cell culture, cell fixation, and immunostaining methods are described in the Supplementary Methods. Quantitative analysis of the nuclear localization of TAZ and pSmad2 was performed as previously described [20]. The detailed methods are described in the Supplementary Methods section.

### 2.2 Ethical approval

All animal testing procedures were performed in accordance with the Declaration of Helsinki and were approved by the Tokyo University of Agriculture and Technology (TUAT) Animal Experimentation Committee (No. 25–60; updated 2019). This study complied with the principles and regulations described in Grundy's editorial (2015) [21].

### 2.3 Bleomycin-induced pulmonary fibrosis mouse model

The fibrosis mouse model and treatment, once-a-day ESM administration, lung tissue removal, sectioning, picrosirius red staining, fibrosis evaluation by Ashcroft methods, and immunofluorescent staining of mouse lung sections are described in the Supplementary Methods. Specifically, a mixture of micronized ESM (Almado, Tokyo, Japan) with jelly was given at a dose of 7.3 mg/kg/day in the BLM + ESM group (equivalent to one dose taken twice daily in a previous study in humans [19] and the amount equivalent to one dose in the current experiment). Jelly (no ESM) was given for the control group and BLM group.

### 2.4 Quantifying collagen content in lung tissue

To estimate the amount of collagen in the lungs, 4-hydroxyproline was quantified using a Hydroxyproline Assay Kit (Sigma-Aldrich, MAK008) according to the manufacturer's protocol. The right lung was used.

### 2.5 Automated histological image analysis of BLM-induced pulmonary fibrosis in mice

Scanning of the picrosirius red-stained lung tissue section slides was performed using a NanoZoomer-XR (Hamamatsu Photonics, Hamamatsu, Japan), and individual lung images were extracted using NDP.view2 software (Hamamatsu Photonics, Hamamatsu, Japan, Ver. 2.9.29). Pulmonary fibrosis density was quantified using open-source ImageJ (Fiji) software (ImageJ Version 1.53t, Java 1.8.0_322 (64 bit)) based on automated thresholds and the production of 2D-reconstituted images according to the literature [22] (Supplementary methods and Supplemental Fig. 1).

### 2.6 Human study subjects and study design

This study was approved by TUAT Ethics Committee (No. 27–09). Written informed consent was obtained from healthy Japanese volunteers. This study was conducted in accordance with the guidelines of the Declaration of Helsinki and those established by the TUAT. After the 8-week trial [19], only the ESM group continued to examine the long-term (22 weeks) effect (aged between 21 and 68, n = 9). Detailed method of human study is described in supplementary method.

## 3 Results

### 3.1 Eggshell membrane, LYZ, and TF increases DCN secretion from fibroblasts

**Fig. 1:**
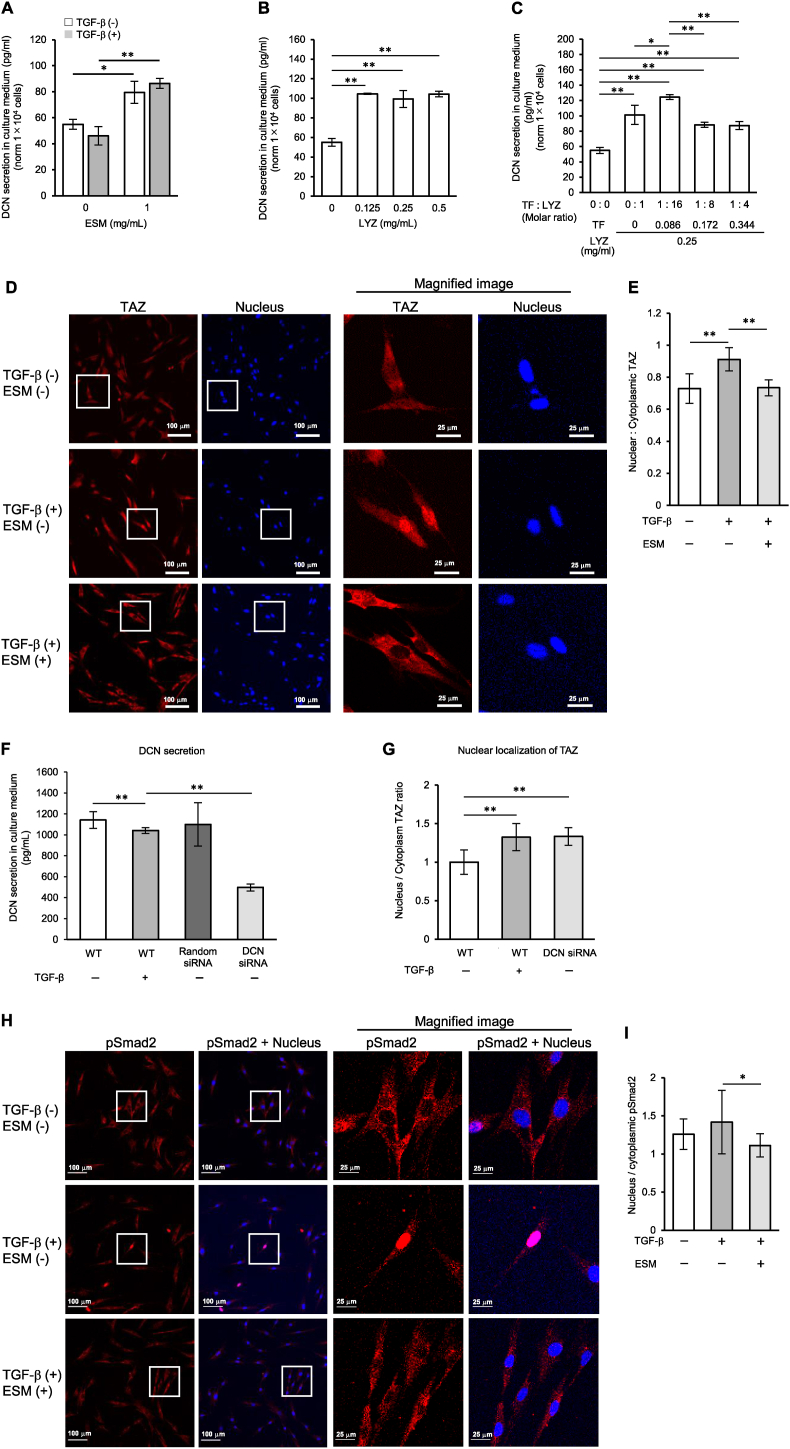
(A–C) Eggshell membrane and its components LYZ and LYZ + TF increase DCN secretion from fibroblasts. Quantifying secreted DCN from human lung fibroblasts WI-38 with or without TGF-β (5 ng/mL) stimulation upon addition of ESM (A), LYZ (B), and LYZ + TF (C) by ELISA (n = 3, *p < 0.05, **p < 0.01). The LYZ in the ESM was about 50 % based on proteomic analysis. The protein content in the hydrolyzed ESM used in (A) was 20 %. Thus, the minimum amount of LYZ added was set at approximately 10 % of 1 mg/ml (A). (D–I) Eggshell membrane treatment inhibited the TGF-β-induced fibrosis pathway in WI-38. Immunofluorescence staining for TAZ with or without TGF-β (5 ng/mL) stimulated WI-38 human lung fibroblasts with or without 1 mg/mL ESM in the medium. E. Quantifying nuclear/cytoplasmic ratios of TAZ staining in WI-38 with or without TGF-β (5 ng/mL) stimulation and with or without ESM (ESM (−): 0 mg/mL and ESM (+): 1 mg/mL). F. Suppressing DCN secretion by *dcn* siRNA to WI-38. N = 3. G. *dcn* knockdown by siRNA causes nuclear localization of TAZ without TGF-β stimulation (n = 15; 3 dish × 5 observations). H. Eggshell membrane treatment inhibited the TGF-β-induced fibrosis pathway in human lung fibroblasts. Immunofluorescence staining for pSmad2 with or without TGF-β (5 ng/mL) stimulated WI-38 human lung fibroblasts stimulated with or without 1 mg/mL ESM in the medium. I. Quantifying nuclear/cytoplasmic ratios of pSmad2 staining in WI-38 stimulated with or without TGF-β (5 ng/mL) in the presence or absence of ESM (ESM (−): 0 mg/mL and ESM (+): 1 mg/mL). DCN, Decorin; ESM, eggshell membrane; LYZ; lysozyme; TF, Ovotransferrin; TGF-β, Transforming growth factor-β.

TGF-β promotes fibrosis and proteoglycan DCN is an antifibrotic agent through binding and neutralization TGF-β. The addition of 1 mg/ml ESM to the medium significantly increased DCN secretion from WI-38 by 1.45-fold (without TGF-β) and 1.87-fold (with TGF-β) compared to that before addition (Fig. 1A). The addition of hydrolyzed LYZ to lung fibroblast culture medium promotes DCN secretion by lung fibroblasts (Fig. 1B). Decorin secretion was highest when the ratio of TF:LYZ was 1:16 (Fig. 1C), corresponding to the ratio present in the ESM extract.

### 3.2 Eggshell membrane treatment inhibited the TGF-β-induced fibrosis pathway in human lung fibroblasts

Nuclear localization of lung fibroblast TAZ was used as an indicator of lung fibroblast activation. TAZ localization to the nucleus significantly increased when WI-38 cells were stimulated with TGF-β (Fig. 1D and E) but was significantly suppressed in the presence of 1 mg/mL ESM (Fig. 1D and E). To examine the involvement of DCN, *dcn*-knockdown cells (*dcn* siRNA) were generated, which showed decreased DCN secretion (Fig. 1F). In the absence of TGF-β, *dcn* knockdown cells (*dcn* siRNA) increased TAZ nuclear localization similar to that by TGF-β stimulation in the absence of TGF-β; therefore, the inhibition of TAZ nuclear localization was DCN-dependent (Fig. 1G). pSmad2 is implicated in pulmonary fibrosis [8]. In this study, TGF-β-stimulated nuclear localization of pSmad2 was suppressed in the presence of ESM (Fig. 1H and I).

### 3.3 Oral administration of ESM ameliorated BLM-induced pulmonary fibrosis in mice

**Fig. 2:**
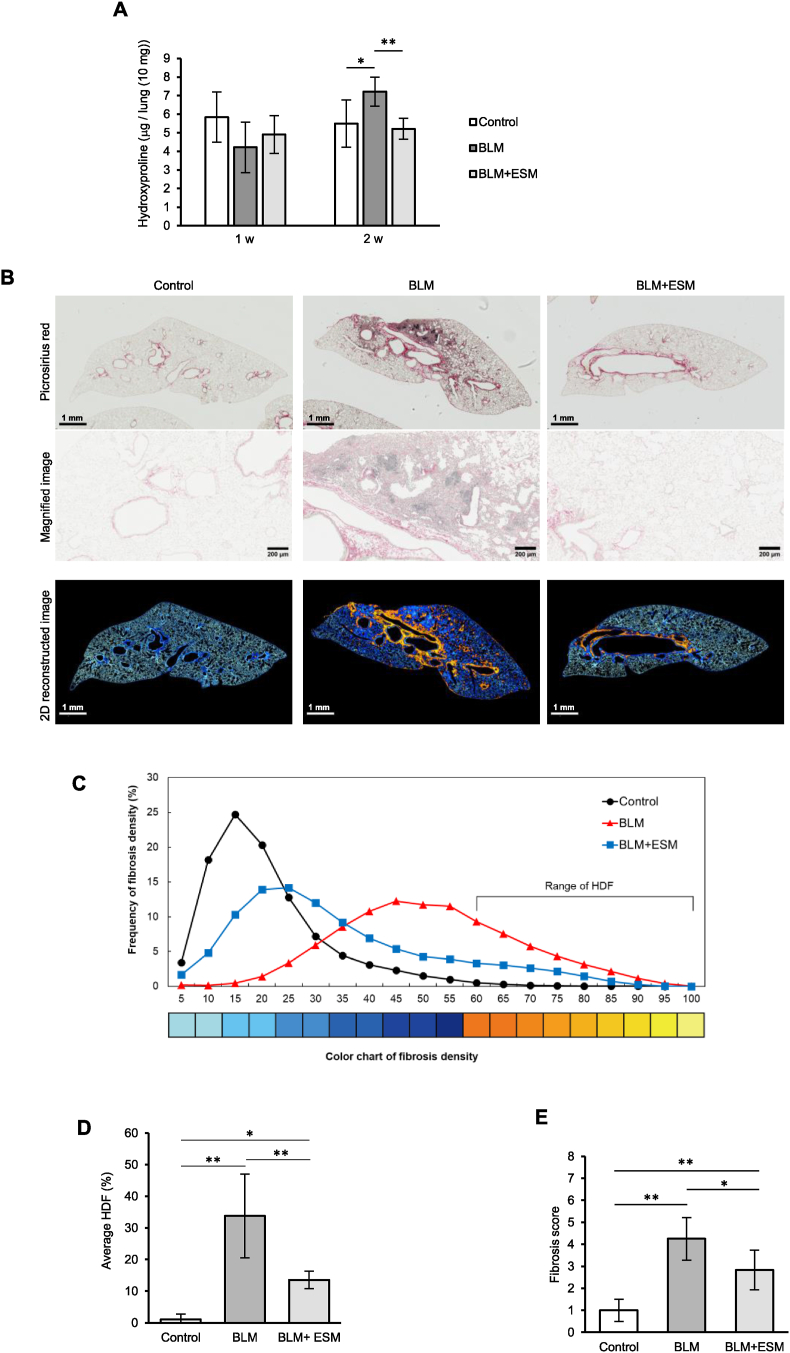
Eggshell membranes alleviated pulmonary fibrosis in a BLM-induced mouse model. A. Quantifying hydroxyproline in the lung. Control: n = 8, BLM: n = 8, BLM + ESM: n = 7. B. Representative images of picrosirius red staining of lung tissue sections (top), magnified image (middle), and 2D reconstructed images corresponding to fibrosis density (bottom). C. The frequency of fibrosis density determined from the classification of the whole unitary fibrosis density values obtained in each lung section. Representative images of each group of 2D reconstructed pulmonary fibrosis density distribution, a characteristic of fibrosis are shown (Fig. 2B lower, Supplemental Fig. 3B, Fig. 2C shows a graph plotting fibrosis density frequency according to the pulmonary fibrosis density color chart. Control n = 8, BLM n = 8, BLM + ESM n = 6 (one sample from the BLM + ESM group was excluded because it was determined as an outlier using the interquartile range) (Supplemental Fig. 2). D. Average high fibrosis density frequency of each group. E. Fibrosis score by the Ashcroft method. Control: n = 8, BLM: n = 8, BLM + ESM: n = 6. BLM, bleomycin; ESM, eggshell membrane; HDF, high-density frequency. (For interpretation of the references to color in this figure legend, the reader is referred to the Web version of this article.)

**Fig. 3:**
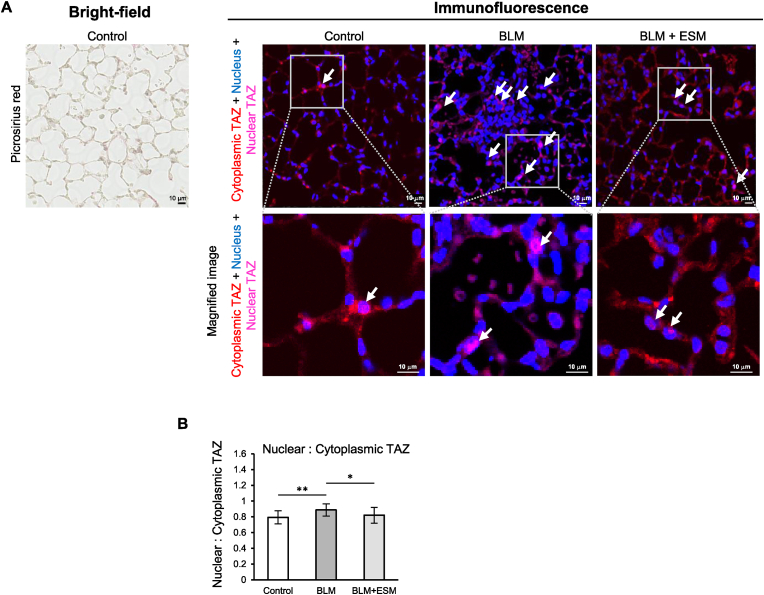
Inhibitory effect of ESM on TAZ nuclear localization in the lung tissue of the BLM mouse model. A. Left: Picrosirius red stained image of a control group lung section. Representative bright-field image of alveoli is shown in the same magnification as in lung tissue sections used for lung immunohistochemistry. Right: Lung immunohistochemistry of merged images of TAZ- and nucleus-staining from each group after two weeks. Only merged images are shown. Red: Cytoplasmic staining by *anti*-TAZ antibody, blue: nucleus, pink: merge of TAZ and nucleus, indicating nuclear localization of TAZ. Arrows point typical TAZ staining in the nucleus. B. Quantifying the nucleus: cytoplasmic ratios of TAZ staining in the lung section. After two weeks of oral intake of ESM, TAZ nuclear localization was significantly reduced in BLM + ESM group compared to the BLM group. Control, BLM (n = 24 (24 fields from 8 slices in 8 animals), BLM + ESM (n = 21 (21 fields from 7 slices in 7 animals)). BLM, bleomycin; ESM, eggshell membrane; TAZ Transcriptional coactivator with PDZ-binding motif. (For interpretation of the references to color in this figure legend, the reader is referred to the Web version of this article.)

Using a mouse model of bleomycin-induced pulmonary fibrosis, the amelioration of ESM to pulmonary fibrosis was evaluated by biochemical quantification, histochemical imaging analyses, and TAZ nuclear localization (TGF-β signaling). After 2 weeks of BLM treatment, BLM + ESM mice showed significantly lower levels of pulmonary hydroxyproline (a collagen deposition marker) than BLM mice (Fig. 2A). Picrosirius red-stained images of the lung sections are shown in the upper part of Fig. 2B (Supplemental Fig. 3A). Collagen deposition and fibrosis were reduced in BLM + ESM mice compared to BLM mice (Fig. 2B, magnified image). The fibrosis density distribution was classified into 20 classes (5, 10, 15, 20, 25, 30, 35, 40, 45, 50, 55, 60, 65, 70, 75, 80, 85, 90, 95, and 100). A pulmonary fibrosis density of 60–100 was defined as a high-density frequency (HDF) characteristic of pulmonary fibrosis (Fig. 2C). The areas of high fibrosis density (orange to yellow, fibrosis density of 60–100) were high in the BLM group, whereas they were lower in the BLM + ESM group (Fig. 2C). The average HDF significantly increased with BLM administration (p < 0.01) and significantly decreased with once-daily ESM intake (p < 0.01) compared with the BLM group (Fig. 2D). The degree of fibrosis assessed by the Ashcroft score was significantly lower in the lungs of BLM + ESM mice than in those of BLM mice after 2 weeks (p < 0.05) (Fig. 2E). Quantification of TAZ staining of lung tissue sections (Fig. 3A) showed that TAZ nuclear localization was significantly reduced in the BLM + ESM group compared to the BLM group (Fig. 3B), which is consistent with the WI-38 results (Fig. 1D and E).

### 3.4 Long-term orally ingested ESM improved respiratory function in humans

ESM supplementation for up to 22 weeks significantly improved respiratory function, VC, and FEV1/FVC (Table 1).

**Table 1:** The effect of oral intake of ESM on respiratory function.

		ESM intake (n = 9)			*p*-value
VC (L)	Before	3.23	±	0.89	0.0002
	Week 22	3.53	±	0.92	
FEV1/FVC (%)	Before	87.50	±	5.26	0.030
	Week 22	90.33	±	3.98	

Pulmonary function tests were performed using a spirometer to investigate the effects of oral ESM intake on pulmonary function. ESM, eggshell membrane; FEV1/FVC; forced expiratory volume in 1 s to forced vital capacity ratio; VC, vital capacity.

## 4 Discussion

Eggshell membrane and its components (LYZ and LYZ + TF) induced DCN secretion in WI-38 human lung fibroblasts. We also verified the inhibition of pSmad2 and transcriptional coactivator TAZ nuclear localization in the presence of the profibrotic cytokine TGF-β using human lung fibroblasts. Oral ingestion of ESM into BLM pulmonary fibrosis model mice significantly reduced the amount of hydroxyproline (an accumulated collagen indicator in fibrosis) and the fibrosis score, and revealed inhibition of elevated TAZ nuclear localization characteristic in lung fibroblasts of patients with IPF. Finally, long-term oral intake of ESM enhanced the respiratory function of the human lung.

In cultured cells, *dcn* overexpression suppressed the biological activity of TGF-β, and simultaneous overexpression of *DCN* suppressed pulmonary fibrosis in TGF-β overexpressing mice [7]. This is the first study showing that ESM stimulates DCN secretion. LYZ and TF are the two major matrix protein components of the mammillary knob with antimicrobial and mineralization activities [23]. The mechanism of the stimulation of DCN secretion from fibroblasts in the presence of LYZ + TF needs to be determined in future studies. LYZ exhibits antitumor- [17], growth inhibition- [24], and antimicrobial activities. This study showed that ESM stimulates endogenous DCN secretion from fibroblasts, which may provide an innovative antifibrotic strategy.

*dcn* is an antifibrotic gene in the lungs [5,6] and DCN suppresses TGF-β-dependent fibrotic signaling [25,26] by binding to and sequestering active TGF-β [7,27,28]. In this study, elevated *dcn* expression from ESM ingestion may have contributed to the suppression of TGF-β-dependent fibrotic signaling, thereby alleviating fibrosis. In pulmonary fibrosis, TGF-β signaling is mediated by YAP/TAZ [8,9]. Crosstalk is suggested between TAZ and other signaling pathways such as TGF-β/Smad [9]. In this study, ESM suppressed the TGF-β-dependent activation of TAZ in lung fibroblasts and BLM mice. The ESM significantly reduced the nuclear localization of pSmad2 in lung fibroblasts. Thus, it is implicated in the inhibition of the TGF-β signaling pathway.

In this study, 22 weeks of ESM supplementation significantly improved VC and FEV1/FVC in humans (Table 1). The application of ESM to human skin increases arm skin elasticity [29] while affecting the dermal papillary layer of mice and inducing *dcn* [29]. After eight weeks of ESM supplementation in humans, subjects demonstrated significantly increased rates of change in arm skin elasticity, zigzag walking speed, and respiratory FEV1/FVC compared to the control group [19]. It is likely that the ingestion of ESM alters the mechanical properties of the lung interstitium and improves respiratory function. ^3^H- labeled ESM was orally administered to mice and was digested and distributed in the blood and various tissues, including the skin and lungs [30]. Thus, orally ingested ESM is expected to affect the lung cells and induce DCN secretion. The respiratory function of the lungs is also affected by changes in the DCN, and mechanics such as lung compliance are degraded in *dcn* knockout mice [31]. These results suggest that ESM may ameliorate lung function by inducing DCN. Detailed signaling and possible mechanism of improvement respiratory function of healthy subjects by ESM are described in the Supplemental Discussions.

In conclusion, ESM enhances the secretion of antifibrosis mediator DCN from fibroblasts, ameliorates BLM-induced pulmonary fibrosis, and suppresses the TGF-β-dependent fibrosis signaling pathway TAZ in vitro and in vivo. This study suggests that the effect of ESM on the lungs is owing to its main components LYZ and TF.

## Data availability

All data generated or analyzed during this study are included in this published article and its Supplementary Information files.

## Fundings

This research was funded by the 10.13039/501100001691Japan Society for the Promotion of Science (grant numbers JP19K11789 and JP 20K11620 a grant-in-aid for Scientific Research.

## CRediT authorship contribution statement

**Eri Ohto-Fujita:** Writing – review & editing, Writing – original draft, Investigation, Funding acquisition, Data curation, Conceptualization. **Miho Shimizu:** Writing – review & editing, Writing – original draft, Visualization, Methodology, Investigation, Funding acquisition, Data curation, Conceptualization. **Aya Atomi:** Investigation. **Hiroki Hiruta:** Investigation. **Ryota Hosoda:** Investigation. **Shinya Horinouchi:** Visualization, Methodology. **Shinya Miyazaki:** Investigation. **Tomoaki Murakami:** Investigation. **Yoshihide Asano:** Conceptualization. **Yukio Hasebe:** Resources. **Yoriko Atomi:** Writing – review & editing, Supervision, Funding acquisition, Conceptualization.

## Declaration of competing interest

The authors declare that they have no known competing financial interests or personal relationships that could have appeared to influence the work reported in this paper.
