# Supplementary material for: Eggshell membrane and its major component lysozyme and ovotransferrin enhance the secretion of decorin as an endogenous antifibrotic mediator from lung fibroblasts and ameliorate bleomycin-induced pulmonary fibrosis
Source: Biochem Biophys Rep. 2024 Aug 12;39:101806. doi: 10.1016/j.bbrep.2024.101806 (PMC11372621; doi:10.1016/j.bbrep.2024.101806)
Supplement: Multimedia component 1 [file mmc1.docx]

**Supplemental file for**

**Eggshell membrane and its major component lysozyme and ovotransferrin enhance the secretion of decorin as an endogenous antifibrotic mediator from lung fibroblasts and ameliorate bleomycin-induced pulmonary fibrosis**

Eri Ohto-Fujita, Miho Shimizu, Aya Atomi, Hiroki Hiruta, Ryota Hosoda, Shinya Horinouchi, Shinya Miyazaki, Tomoaki Murakami, Yoshihide Asano, Yukio Hasebe, Yoriko Atomi*

**Table of Contents**

**Supplemental Figure S1.** Overview of the image analysis

**Supplemental Figure S2.** Determination of an outlier

**Supplemental Figure S3.** All histochemical lung images and their 2D-reconstructed images from 2 weeks control, BLM treatment, BLM+ESM mice

**Supplemental Methods**

**Supplemental Discussions**

**Supplemental References**

****Supplemental Fig. 1

**Supplemental Fig.1** Overview of the image analysis

Construction of 2D-reconstituted fibrosis density image (A). Procedures are described in the Supplementary method. ROI of a scanned color image of picrosirius-red stained lung slice (Aa). Separate into HSB stack, extract by saturation channel and background subtraction by 50 px rolling ball radius (Ab). Binarized image of Collagen deposited area (Ac) and its 49 x 49 kernel mean filtered image (Ad). Binarized image of Lung area (Ae) and its 49 x 49 kernel mean filtered image (Af). 2D-reconstituted image of fibrosis density (Ag). Visual check of the ROI of binarized images of the Lung and collagen deposited area (B). ROI of scanned image (Ba). Overlay of ROI (pink line) based on the binarized image of the Lung area over the scanned image (Bb). Overlay of ROI (blue line) based on the binarized image of collagen deposited area over the scanned image (Bc). Overlay of the binarized image of the collagen deposited area (blue ROI) over the binarized image of the Lung area (pink ROI) (Bd).

Supplemental Fig. 2

**Supplemental Fig.2** Determination of an outlier

Box-and-whisker plot of mean fibrosis density (%) of control (n=8), BLM (n=8), and BLM+ESM (n=7) after two weaks of BLM treatment. Outliers here are defined as observations that fall below the first quartile (Q1 )− 1.5 interquartile (IQR) or above the third quartile (Q3) + 1.5 IQR. One of the BLM+ESM lung was dertermined as an outlier from this box plot.

Supplemental Fig. 3

**Supplemental Fig. 3** All histochemical lung images and their 2D-reconstructed images from 2 weeks control, BLM treatment, BLM+ESM mice

Picrosirius red-stained images (A) and their 2D-reconstructed images (B) of lung section in each group. The bar is 1 mm.

**Supplementary methods**

***In vitro cell studies***

**Cell culture**

WI-38 lung fibroblasts (ATCC CCL-75) were obtained from ATCC and maintained in MEM/EBSS (Hyclone Laboratories Inc., Logan, UT, USA) supplemented with 10% fetal bovine serum (NICHIREI BIOSCIENCES INC., Tokyo, JAPAN, sodium pyruvate (Gibco, Waltham, MA, USA), NEAA (Gibco, Waltham, MA, USA), and PSN (Gibco, Waltham, MA).

**Cell fixation and immunostaining**

Cells were fixed with neutral buffered formalin solution, (HT5011 formalin solution 10% neutral buffer, Sigma-aldrich, St. Louis, MO, USA) and incubated with anti-TAZ antibody produced in rabbits (HPA 007415; SIGMA, St. Louis, MO, USA) or anti phospho-SMAD2 antibody produced in rabbits (#3108, Cell Signaling Technology, Inc., Danvers, MA, USA). This was followed by incubation with anti-rabbit Alexa plus555 antibody (A32732; Invitrogen, Carlsbad, CA, USA) and Hoechst33325 (Invitrogen, Carlsbad, CA, USA) for DNA.

**Determination of DCN in culture medium in DCN knockdown WI-38**

WI-38 cells (passage 4-7, 2 × 10^4^ cells) were seeded overnight on 22 × 22 mm^2^ coverslips. Medium was replaced with 2 mL fresh medium without serum for treatment with TGF-β1 or siRNA DCN, WI-38 cells were treated with rhTGF-β (5 ng/mL) or siRNA DCN (13 nM) (predesign siRNA ID: SASI_Hs01_00191017, Sigma-Aldrich) or random siRNA (13 nM) (SIC001, MISSION® siRNA Universal Negative Control #1, Sigma-Aldrich) for 24 hours. siRNA was transfected with Lipofectamine™ RNAiMAX Transfection Reagent (#13778075, Thermo Fisher Scientific). Before fixing the cells, the culture supernatant was collected and DCN in the medium was quantified using Decorin DuoSet ELISA.

**Quantification of nuclear localization of TAZ and pSmad**

Quantitative analysis of nuclear localization was performed in accordance with a previous report [1]. Fluorescence images were taken with a Nikon A1RMP confocal microscope. Image analysis was performed using the ImageJ software (version 1.48). Five fields from each strain were selected for analysis. For each field, binary image masks of TAZ or F-actin (for pSmad2) and Hoechst33342 positive staining were created to define a region of interest (ROI) for analysis. This was applied to a median filter (3 × 3 pixel radius) to remove noise. The image was then converted to a binary mask using the automated thresholding of the Isodata algorithm. The nuclear ROI was defined using Hoechst 33342 staining mask. The nuclear ROI was defined using an image calculator, the Hoechst33342 mask was subtracted from the TAZ mask or F-actin (for pSmad2) mask to create a staining mask defining the cytoplasmic ROI. Each ROI mask was then applied to the original TAZ or pSmad2 (Alexa Plus 555) stained images, and the image calculator was used to separate nuclear and cytoplasmic staining within each field. Quantitative fluorescence data were exported to Microsoft Excel using the histograms generated in ImageJ. Nuclear and cytoplasmic staining intensities were compared to determine the nuclear/cytoplasmic ratio as a relative measure of TAZ or pSmad2 nuclear localization.

***In vivo* animal studies**

**Fibrosis mouse model and treatment**

This experiment involved 48 C57BL/6 mice (female, 8 weeks old) purchased from Sankyo Laboratory Services, Inc. (Tokyo, Japan), which were divided the control group (n = 16), BLM group (n = 16), and BLM+ESM group (n = 16). Due to their susceptibility, C57BL/6 mice are often used to study pulmonary fibrosis [2]. Mice were maintained in a room with controlled temperature (18°C–22°C) and humidity (50%–70%), given water and solid feed (Rodent Diet CE-2, CLEA Japan, Inc., Tokyo, Japan), and exposed to alternating 12-hr light/dark cycles.

After 1 week of pre-rearing, BLM (3 mg/kg; Nippon Kayaku Co. Ltd., Tokyo, Japan) for BLM group and BLM+ESM group or PBS for control group was intratracheally administered, while the animals were anesthetized with medetomidine (Nippon Zenyaku Kogyo, Fukushima, Japan), midazolam (Sandoz Inc., Tokyo, Japan), and butorphanol (Meiji Seika Co., Ltd., Tokyo, Japan); Nippon Kayaku Co., Tokyo, Japan) was administered intratracheally (Day 0). Immediately thereafter, the mice were force-fed ESM containing jelly (MediGel Sucralose, Clear H_2_O, ME, USA) for BLM+ESM group or jelly for control and BLM group by sonde, and medetomidine antagonists (Antisedan, Nippon Zenyaku Kogyo, Fukushima, Japan) were administered intraperitoneally (Day 0). The mice were then returned to the cage and allowed to recover from anesthesia. The following day, ESM was orally administered once daily for 1 or 2 weeks. Specifically, a mixture of micronized ESM (Almado, Tokyo, Japan) with jelly was given at a dose of 7.3 mg/kg/day in the BLM+ESM group (equivalent to one dose taken twice daily in a previous study in humans (reference [20] in the manuscript) and the amount equivalent to one dose in the current experiment). Jelly (no ESM) was given for the control group and BLM group. Water and solid feed (Rodent Diet CE-2, CLEA Japan, Inc., Tokyo, Japan) were provided ad libitum. Mice were weighed once daily.

**Lung tissue resection**

After 1 week, 24 mice (control: n = 8, BLM: n = 8, BLM+ESM: n = 8) were deeply anesthetized by 5.0% isoflurane inhalation in an induction box followed by maintained by 2.0% isoflurane with an anesthetic mask. Afterwards, the abdomen was opened, blood was drawn from the abdominal vena cava, and the animals were sacrificed to remove the lungs. The lungs and bronchi were exposed, and 1 ml of PBS was injected through the bronchioles for bronchoalveolar lavage. After 2 weeks, the remaining mice (control: n = 8, BLM: n =8, BLM+ESM: n = 7 (One of BLM+ESM group died before dissection on day 14.)) were anesthetized as above, dissected, and lungs were removed.

**Lung section**

After ligation and removal of the right lung, neutral buffered formalin (HT5011 formalin solution 10% neutral buffer, Sigma-Aldrich, St. Louis, MO, USA) was slowly injected into the left lung. The excised left lung was fixed overnight in neutral buffered formalin dehydrated, and paraffin embedded. Paraffin blocks were sliced into 4-μm sections.

**Picrosirius Red staining**

Lung sections were stained with Picrosirius Red (Polysciences, Inc.).

**Automated histological image analysis of BLM-induced pulmonary fibrosis in mice**

For quantification of pulmonary fibrosis density by automated thresholds and production of 2D-reconstituted image, open-source ImageJ (Fiji) software (ImageJ Version 1.53t, Java 1.8.0_322 (64 bit)) was used. The procedures are summarized in Supplementary Figure 1. For clarity, 170 x 170 μm^2^ ROI is shown. Briefly, starting from a scanned color image (tiff) of a picrosirius-red stained lung slice (Supplementary figure 1Aa), the lung area was separated into HSB stack, extracted by saturation channel, and the background was subtracted by 50 px rolling ball radius (Supplementary figure 1Ab). The collagen deposited area and lung area were extracted by 2 different automatic threshold tools, one using the Otsu [3] (Supplementary Figure 1Ac), and another using the Mean followed by denoise by analyze particles (Supplementary Figure 1Ae). To construct a 2D image, a 49 x 49 kernel mean filter (55.4 x 55.4 μm^2^ in our study) was used (Supplementary Figure 1Ad and f). To quantify the fibrosis density, the collagen deposited area was divided by the lung area (Supplementary Figure 1Ad). To check the selected filter sets for collagen deposited area and lung area were appropriate, several overlay images were constructed for a 170 x 170 μm^2^ ROI as shown in (Supplementary Figure 1B).

To quantify the distribution of pulmonary fibrosis density, the fibrosis density distribution was classified into 20 classes (5, 10, 15, 20, 25, 30, 35, 40, 45, 50, 55, 60, 65, 70, 75, 80, 85, 90, 95, 100). The frequency of fibrosis density was calculated by dividing the fibrosis density value of a particular class by the total number of density values in all 20 classes. To visualize the distribution of fibrosis density, a 2D reconstructed image was constructed by assigning a pseudocolor to the fibrosis density according to the classification. The frequency of fibrosis density was determined from the classification of all unit density values obtained in each lung section. The high fibrosis density frequency (HDF) corresponds to the sum of the frequencies of high fibrosis densities (**60-100**) restricted to fibrotic changes. Average HDF of each group (Fig. 2D) was graphed.

**Fibrosis evaluation by Ashcroft method.**

Pulmonary fibrosis scores from picrosirius red-stained paraffin sections of the lung were determined using a blinded Ashcroft scoring system [3]. Pulmonary fibrosis was scored according to the following histological features:0: normal lung; 1: minimal fibrotic thickening of alveolar or bronchiolar walls; 2: intermediate grade between 1 and 3; 3: moderate wall thickening without obvious damage to lung structure; 4: intermediate grade between 3 and 5; 5: increased fibrosis with obvious damage to lung structure and formation of fibrous bands or small fibrous masses; 6: intermediate grade between 5 and 7; 7: highly distorted structure with large fibrotic areas (including " honeycomb lung"); 8: complete fibrotic loss of the lung field.

**Immunofluorescence of mouse lung sections**

Mouse lung sections were fixed in formalin and incubated with the following primary antibodies: anti-TAZ antibody (1:50; HPA 007415; SIGMA, St. Louis, MO, USA), followed by the secondary antibody anti-rabbit IgG Alexa Fluor 546 (1:50; Cat# A10040; Invitrogen, Carlsbad, CA, USA). The cell nuclei were stained with Hoechst 33342. Immunostained sections were observed under a Nikon A1 RMP confocal microscope (Nikon Corp., Tokyo, Japan). Quantitative analysis of the nuclear localization of TAZ was performed according to previous reports [1], as was the analysis of lung fibroblasts.

**Eggshell membrane human supplementation methods and pulmonary function tests**

Eggshell membrane (Almado, Tokyo, Japan) supplementation (4 tablets in the morning and evening) and pulmonary function tests were performed as previously described [12].

**Statistical analysis**

Data were expressed as the mean ± standard deviation (SD). Data were evaluated using analysis of variance and Tukey's post-hoc analysis or an unpaired t-test. Statistical significance was set at p < 0.05.

**Supplemental Discussions**

**Why nuclear localization of lung fibroblast TAZ was used as an indicator of lung fibroblast activation in this study**

We chose TAZ as an indicator mainly from following reasons. First, because previous research has investigated the involvement of TAZ, a mechanical transducer, in pulmonary fibrosis [4]. Second, increased lung compliance in *dcn* KO mice, reflecting reduced elastic contractility of the alveoli [5]. Third, enhanced secretion of DCN from WI-38 cells and TGF-β induced TAZ nuclear translocation was relatively fast (5 hr) in our study compared to TGF-β induced fibroblast activation, transition to myofibroblast, as indicated by a production of α-SMA (>24 hr).

**YAP or TAZ signaling in Pulmonary fibrosis**

Different regulatory and downstream functions of YAP and TAZ have been reported in the Hippo pathway, which regulates tissue homeostasis [6]. Furthermore, in the context of skin fibrosis, the activation of YAP via tubulin acetylation on ECM stiffness has also been reported to drive fibroblast-myofibroblast transition [7]. In future studies, investigating how YAP is involved in ESM-aid reduction of fibrotic signaling will clarify the specific cascade which enhance lung function in healthy human subjects.

**Role of SMADS in the suppression of lung fibrosis by ESM**

TGF-β1 induces phosphorylation of Smad2/3, and nuclear translocation of phosphorylated Smad2/3 induces transcription of fibrosis-promoting genes in various tissues, including lung, liver, and kidney [8]. In most cases, two molecules of SMAD2 or SMAD3 form a heterotrimer in complex with SMAD4 before translocation to the nucleus, where they function as co-regulators of gene expression [9]. On the other hand, the PDL1 mediates lung fibroblasts paper [10], which shows that PDL1 is involved in the enhancement of pSmad3 in IPF, only examined pSmad3 and reported a specific pathway. However, we have not examined this in WI-38 lung fibroblasts, and it would be interesting to investigate the involvement of pSmad3 in WI38 lung fibroblasts in the future. Future studies are beginning to show that the TGF-β-dependent fibroblast to myofibroblast transition is suppressed in the presence of eggshell membrane, and we would like to examine what differentiation signals are involved, including PDL1. The involvement of SMAD7, which suppresses TGF-b signaling [11] in ESM-induced fibrosis amelioration is open question for future study.

**Improvement of respiratory function in healthy subjects by supplementation with ESM.**

In Figure 1, the data show that ESM administration to lung fibroblasts increased decorin secretion without activation stimulation by TGF-β. In other words, activation of TGF-β is not necessary for increased secretion of decorin by ESM on lung fibroblasts. Therefore, we consider that ESMs may increase decorin secretion in the lungs even in healthy individuals. In fact, we have already shown in a previous report [12] that the FEV1/FVC (forced expiratory volume in 1 s to forced vital capacity ratio significantly increased after 8 weeks of ESM intake in healthy subjects in comparison with a control group. Although preliminarily, we have observed that ESM was orally administered to healthy B6 mice as a mouse experiment and that decorin gene expression was significantly increased. As to why ESM supplementation improved respiratory function in healthy subjects, even though no fibroblast activation was observed in healthy subjects, we consider the following. Although the effect of aging on decorin is not clear in the lungs, the GAG chain size of decorin is smaller in older skin than in younger skin due to aging, and age-related changes in decorin GAGs contribute to skin fragility in the elderly [13]. The effects of decreasing decorin in the lungs are evident from studies of KO mice [5], which show that the mechanical properties of the lungs are altered in decorin KO mice (increased lung compliance = decreased elastic contractility of alveoli). Thus, an increase in decorin in the lungs may improve the mechanical property of the lungs. On the other hand, since transient expression of decorin in the lungs reduces bleomycin pulmonary fibrosis [14], it is likely that ESM ingestion promotes decorin expression, which is altered with aging, contributing to improved lung and respiratory function during aging.

**Supplemental References**

[1] M. Noursadeghi, J. Tsang, T. Haustein, R.F. Miller, B.M. Chain, D.R. Katz, Quantitative imaging assay for NF-kappaB nuclear translocation in primary human macrophages. J Immunol Methods. 329 (2008) 194-200, <https://doi.org/10.1016/j.jim.2007.10.015>.

[2] L. Walkin, S.E. Herrick, A. Summers, P.E. Brenchley, C.M. Hoff, R. Korstanje, P.J. Margetts, The role of mouse strain differences in the susceptibility to fibrosis: a systematic review. Fibrogenesis Tissue Repair. 6 (2013) 18, <https://doi.org/10.1186/1755-1536-6-18>.

[3] T. Ashcroft, J.M. Simpson, V. Timbrell, Simple method of estimating severity of pulmonary fibrosis on a numerical scale. J Clin Pathol. 41 (1988) 467-470, <https://doi.org/10.1136/jcp.41.4.467>.

[4] S. Noguchi, A. Saito, Y. Mikami, H. Urushiyama, M. Horie, H. Matsuzaki, H. Takeshima, K. Makita, N. Miyashita, A. Mitani, T. Jo, Y. Yamauchi, Y. Terasaki, T. Nagase, TAZ contributes to pulmonary fibrosis by activating profibrotic functions of lung fibroblasts. Sci Rep. 7 (2017) 42595, <https://doi.org/10.1038/srep42595>.

[5] A. Fust, F. LeBellego, R.V. Iozzo, P.J. Roughley, M.S. Ludwig, Alterations in lung mechanics in decorin-deficient mice. Am J Physiol Lung Cell Mol Physiol. 288 (2005) L159-166, <https://doi.org/10.1152/ajplung.00089.2004>.

[6] S.W. Plouffe, K.C. Lin, J.L. Moore, 3rd, F.E. Tan, S. Ma, Z. Ye, Y. Qiu, B. Ren, K.L. Guan, The Hippo pathway effector proteins YAP and TAZ have both distinct and overlapping functions in the cell. J Biol Chem. 293 (2018) 11230-11240, <https://doi.org/10.1074/jbc.RA118.002715>.

[7] D. Wen, Y. Gao, Y. Liu, C. Ho, J. Sun, L. Huang, Y. Liu, Q. Li, Y. Zhang, Matrix stiffness-induced alpha-tubulin acetylation is required for skin fibrosis formation through activation of Yes-associated protein. MedComm (2020). 4 (2023) e319, <https://doi.org/10.1002/mco2.319>.

[8] A. Futakuchi, T. Inoue, F.Y. Wei, M. Inoue-Mochita, T. Fujimoto, K. Tomizawa, H. Tanihara, YAP/TAZ Are Essential for TGF-beta2-Mediated Conjunctival Fibrosis. Invest Ophthalmol Vis Sci. 59 (2018) 3069-3078, <https://doi.org/10.1167/iovs.18-24258>.

[9] J.H. Kang, M.Y. Jung, X. Yin, M. Andrianifahanana, D.M. Hernandez, E.B. Leof, Cell-penetrating peptides selectively targeting SMAD3 inhibit profibrotic TGF-beta signaling. J Clin Invest. 127 (2017) 2541-2554, <https://doi.org/10.1172/JCI88696>.

[10] X. Guo, C. Sunil, O. Adeyanju, A. Parker, S. Huang, M. Ikebe, T.A. Tucker, S. Idell, G. Qian, PD-L1 mediates lung fibroblast to myofibroblast transition through Smad3 and beta-catenin signaling pathways. Sci Rep. 12 (2022) 3053, <https://doi.org/10.1038/s41598-022-07044-3>.

[11] X. Yan, Y.G. Chen, Smad7: not only a regulator, but also a cross-talk mediator of TGF-beta signalling. Biochem J. 434 (2011) 1-10, <https://doi.org/10.1042/BJ20101827>.

[12] E. Ohto-Fujita, N. Hatakeyama, A. Atomi, S. Yasuda, S. Kodama, T. Atomi, K. Tanaka, N. Hirose, K. Harada, Y. Asano, T. Watanabe, Y. Hasebe, M. Shimizu, Y. Atomi, Effect of Eggshell Membrane Powder Intake on the Body Function of Healthy Individuals. Journal of Fiber Science and Technology. 77 (2021) 258-265

, <https://doi.org/10.2115/fiberst.2021-0029>.

[13] Y. Li, Y. Liu, W. Xia, D. Lei, J.J. Voorhees, G.J. Fisher, Age-dependent alterations of decorin glycosaminoglycans in human skin. Sci Rep. 3 (2013) 2422, <https://doi.org/10.1038/srep02422>.

[14] M. Kolb, P.J. Margetts, T. Galt, P.J. Sime, Z. Xing, M. Schmidt, J. Gauldie, Transient transgene expression of decorin in the lung reduces the fibrotic response to bleomycin. Am J Respir Crit Care Med. 163 (2001) 770-777, <https://doi.org/10.1164/ajrccm.163.3.2006084>.
